# Cyclase-associated protein 2 (CAP2) controls MRTF-A localization and SRF activity in mouse embryonic fibroblasts

**DOI:** 10.1038/s41598-021-84213-w

**Published:** 2021-02-26

**Authors:** Lara-Jane Kepser, Sharof Khudayberdiev, Laura Soto Hinojosa, Chiara Macchi, Massimiliano Ruscica, Elena Marcello, Carsten Culmsee, Robert Grosse, Marco B. Rust

**Affiliations:** 1 Institute of Physiological Chemistry, Philipps-University of Marburg, 35032 Marburg, Germany; 2 DFG Research Training Group, Membrane Plasticity in Tissue Development and Remodeling, GRK 2213, Philipps-University of Marburg, 35032 Marburg, Germany; 3 Institute of Pharmacology, University of Marburg, 35032 Marburg, Germany; 4 Department of Pharmacological and Biomolecular Sciences, University of Milan, 20133 Milan, Italy; 5 Institute of Pharmacology and Clinical Pharmacy, University of Marburg, 35032 Marburg, Germany; 6 Center for Mind, Brain and Behavior (CMBB), University of Marburg and Justus-Liebig-University Giessen, Hans-Meerwein-Strasse 6, 35032 Marburg, Germany

**Keywords:** Actin, Transcription, Cytoskeleton

## Abstract

Recent studies identified cyclase-associated proteins (CAPs) as important regulators of actin dynamics that control assembly and disassembly of actin filaments (F-actin). While these studies significantly advanced our knowledge of their molecular functions, the physiological relevance of CAPs largely remained elusive. Gene targeting in mice implicated CAP2 in heart physiology and skeletal muscle development. Heart defects in CAP2 mutant mice were associated with altered activity of serum response factor (SRF), a transcription factor involved in multiple biological processes including heart function, but also skeletal muscle development. By exploiting mouse embryonic fibroblasts (MEFs) from CAP2 mutant mice, we aimed at deciphering the CAP2-dependent mechanism relevant for SRF activity. Reporter assays and mRNA quantification by qPCR revealed reduced SRF-dependent gene expression in mutant MEFs. Reduced SRF activity in CAP2 mutant MEFs was associated with altered actin turnover, a shift in the actin equilibrium towards monomeric actin (G-actin) as well as and reduced nuclear levels of myocardin-related transcription factor A (MRTF-A), a transcriptional SRF coactivator that is shuttled out of the nucleus and, hence, inhibited upon G-actin binding. Moreover, pharmacological actin manipulation with jasplakinolide restored MRTF-A distribution in mutant MEFs. Our data are in line with a model in which CAP2 controls the MRTF-SRF pathway in an actin-dependent manner. While MRTF-A localization and SRF activity was impaired under basal conditions, serum stimulation induced nuclear MRTF-A translocation and SRF activity in mutant MEFs similar to controls. In summary, our data revealed that in MEFs CAP2 controls basal MRTF-A localization and SRF activity, while it was dispensable for serum-induced nuclear MRTF-A translocation and SRF stimulation.

## Introduction

Cyclase-associated proteins (CAPs) have been recognized as actin-binding proteins (ABP) two decades ago^1–4^, but significant progress in their molecular function has been achieved only recently^5–11^. These studies unraveled a role for yeast and mammalian CAPs in disassembly of actin filaments (F-actin) and in the ATP-for-ADP-exchange on actin monomers (G-actin) that is essential for F-actin assembly. Hence, CAPs emerged as important regulators of F-actin dynamics, the spatiotemporally controlled assembly and disassembly of F-actin^12^. While these studies advanced our knowledge of their molecular functions, the physiological relevance of mammalian CAPs largely remained elusive, also because appropriate animal models were lacking. This holds true specifically for the ubiquitously expressed family member CAP1^13^, while recent studies revealed arrhythmia, cardiac conduction defects as well as dilated cardiomyopathy in systemic and heart-specific CAP2 mutant mice^14–16^. Additionally, skeletal muscle development and myofibril differentiation was retarded in systemic CAP2 mutants, which displayed a myopathy characterized by a large number of ring fibers associated with motor function deficits^17^. Together, these studies emphasized a pivotal role for CAP2 in striated muscles, in agreement with its abundant expression in heart and skeletal muscle^14,17,18^. Heart defects in CAP2 mutant mice were associated with altered gene expression including an upregulation of genes whose expression is controlled by serum response factor (SRF), and they were partially restored upon pharmacological SRF inhibition^19^, suggesting a causal relationship between SRF dysregulation and heart pathology. However, the mechanism underlying increased SRF activity upon CAP2 inactivation remained unknown.

SRF is a ubiquitously expressed and highly conserved transcription factor that was first identified in studies of fibroblast serum response. Its activity is mainly regulated by two classes of transcriptional coactivators, namely the ternary complex factors (TCFs) as well as myocardin and myocardin-related transcription factors (MRTFs)^20^. TCFs and MRTFs compete for a common surface domain on the DNA-binding domain of SRF, interact with SRF in a mutually exclusive manner and activate different sets of SRF-target genes^21,22^. The TCF-SRF pathway is promoted by Ras and mitogen-activated protein kinases (MAPKs), induces expression primarily of immediate early genes (IEG) and has been implicated in cell-cycle re-entry and proliferation^22^. Instead, the MRTF-SRF pathway is controlled by the availability of G-actin and induces expression of cytoskeleton-related genes^21^. Specifically, G-actin binds to MRTF and promotes its translocation into the cytosol^23^, thereby inhibiting MRTF-SRF-dependent gene expression^20^. Hence, the actin regulator CAP2 may control SRF activity via a mechanism that involves actin and MRTF.

By exploiting mouse embryonic fibroblasts (MEFs) from systemic CAP2 mutant mice, we here (i) tested whether CAP2 controls SRF activity in cell types other than heart cells and (ii) aimed at deciphering the underlying mechanism. Reporter assays and quantitative PCR (qPCR) revealed reduced SRF activity in MEFs upon CAP2 inactivation. Impaired SRF activity in CAP2 mutant MEFs was associated with altered actin turnover, a shift in the actin equilibrium towards G-actin and reduced nuclear MRTF-A levels. Further, MRTF-A distribution in mutant MEFs was normalized by pharmacological actin manipulation. While our data were in line with a role for CAP2 in regulating SRF activity via the actin-MRTF-A pathway in non-stimulated MEFs, serum stimulation equally induced nuclear MRTF-A translocation and SRF activity in control and CAP2 mutant MEFs.

## Material and methods

### Preparation and culture of MEFs

MEFs were isolated from CAP2-KO mice and CTR littermates at E12.5 as previously described^24,25^. Briefly, dissected embryos were minced and treated with 0.25% trypsin (Invitrogen) after removal of the head and organs. After 15 min of incubation at 37 °C, tissue was pulled through a 0.9-mm needle to obtain isolated cells. After washing twice with serum-free Dulbecco’s modified Eagle’s medium (DMEM; Invitrogen), isolated cells were cultured in DMEM containing 10% fetal calf serum (FCS; PAA). Primary MEFs (termed passage 0) were grown for 1 day. On the next day, MEF immortalization was started according to the 3-day transfer protocol (3T3) immortalization protocol that defined immortalization as completed after passage 12. MEFs were kept at 37 °C and 5% CO_2_ in DMEM containing 4.5 g/l glucose and 10% FCS, as well as penicillin (100 m/mL; PAA) and streptomycin (100 mg/mL; PAA). MEFs were passaged every 2–3 days. All experiments were performed with MEFs of P12–P40. All experiments were carried out in accordance to relevant guidelines and regulations. Killing of mice was approved by internal animal welfare authorities of the University of Marburg and the Regierungspräsidium Giessen (reference: AK-6-2014-Rust).

### Generation of MEF cell line stably expressing MRTF-A-GFP

MRTF-A-GFP was stably expressed in MEF cells (control and CAP2 mutant cell lines). For that, first HEK293T cells were transfected using the calcium phosphate method. For lentivirus production, HEKT293T cells were cotransfected with the lentiviral packaging vectors psPAX and pMDG.2 together with the pInducer-MRTF-A GFP plasmid as described before^26^. After 48 h, supernatants containing viral particles were harvested, filtered, and used to transduce MEF cells. Transduced MEF cells were selected by FACS-based cell sorting. Expression of MRTF-A–GFP from pInducer20 was induced by 333 ng/mL doxycycline.

### Live cell imaging

To analyze MRTF-A subcellular localization upon serum stimulation, MEF cells stably expressing pIND20-MRTF-A-GFP were serum-deprived for 48 h and then live cell imaging was performed. Cells were stimulated 24 h before imaging with 333 ng/mL doxycycline to induce MRTF-A-GFP expression. Serum stimulation was performed by adding 20% serum directly to the cells under the microscope to follow the effects of the stimulation on MRTF-A translocation over time. Microscopic imaging was performed using confocal laser-scanning microscope (LSM 800, Carl Zeiss) and a 63 × 1.4 NA oil objective lens (Carl Zeiss). Time-lapse microscopy was performed at 37 °C in a CO_2_-humidified incubation chamber (Pecon, CO_2_, module S1) using ZEN software (Carl Zeiss).

### Fluorescence recovery after photobleaching (FRAP)

MEFs were plated on 35 mm WillCo-dish Glass Bottom Dishes coated with 0.01% calf skin collagen (Sigma Aldrich) in 0.1 M acetic acid. Overexpression of eGFP-actin was performed by overnight transfection of pEGFP-C1-Actin (kindly provided by Kristin Michaelsen-Preusse) using Lipofectamine2000 in serum- and antibiotic-free DMEM medium. After exchanging the medium to the full DMEM, cells were cultured for 48 h. FRAP imaging was carried out with a Leica TCS SP5 II (FRAP-Wizard) confocal laser scanning microscope equipped with a temperature-controlled chamber. MEFs were imaged with 63 × (+ 6 zoom) objective at 35 °C in 1 × HBSS (supplemented with 4 mM NaCO_3_ and 2 mM CaCl_2_). The following imaging settings have been applied: 512 × 512 format, speed 700 Hz, 2-line averaging, pinhole AU 3, 50% of argon laser power (~ 25 mW luminous power in focal plane), bleaching with 100% and image acquisition 3–7% power intensity of AOTF 488 nm (FRAP-wizard). Imaging/bleaching program: pre-bleaching 5 × 2 s, bleaching 3 × 1.5 s (ROI 5 µM diameter), post-bleaching 10 × 2 s, 15 × 5 s.

The image series were analyzed using FIJI software according to previous studies^27^. In brief, the background and bleaching correction was applied, and then normalized fluorescence intensity for each time point was calculated. Nonlinear curve fitting (one phase exponential association) of the fluorescence intensity was performed with GraphPad Prism, where the net recovery after photobleaching is provided by the following equation: Y = Y0 + (Plateau − Y0) × (1 − exp(− K × x)), where Y0 is the Y value when time is zero directly after the bleaching impulse, Plateau is the Y value at infinite times, expressed as a fraction of the fluorescence before bleaching and was used to determine the dynamic actin pool (F-actin dynamic). The stable pool (F-actin stable) is the fraction of fluorescence that does not recover within the imaging period of 95 s calculated as 1 − (F-actin dynamic), K is the rate constant, and τ is the time constant, expressed in seconds, it is computed as the reciprocal of K.

### SRF-luciferase reporter gene assay

To assess SRF activity, we generated MEF cell lines (control and CAP2 mutant) expressing the firefly luciferase reporter where MRTF-SRF promoter 3 Da.luc was linked to GFP. To generate the MEF cell lines stably expressing the MRTF-SRF luciferase reporter, first HEK293T cells were transfected using the calcium phosphate method. For lentivirus production, HEKT293T cells were cotransfected with the lentiviral packaging vectors psPAX and pMDG.2 together with the lentiviral vector FUGW expressing MRTF-SRF promoter 3 Da.luc linked to GFP. Generation of the lentiviral luciferase reporter construct has been described before^26^. After 48 h, supernatants containing viral particles were harvested, filtered, and used to transduce MEF cells. Transduced MEF cells were selected by FACS-based cell sorting.

MEF cells expressing the MRTF-SRF luciferase reporter were serum-deprived overnight and stimulated either with or without 20% serum for 24 h or 48 h. Then, cells were lysed with 200 μL Triton lysis buffer on ice and collected in 1.5 mL Eppendorf tube, followed by 10 min centrifugation at 13,000 rpm at 4 °C. The amount of firefly luciferase was measured luminometrically for each condition using a Glomax 96 Microplate Luminometer (Promega)^28^.

### Nucleofection

To study MEF morphology and CAP2 localization, MEF cells were transfected with either 3 µg pEGFP-C1 (Clontech) or 3 µg pEGFP-C1-MmCAP2 (kindly provided by Elena Marcello) using the 4D Nucleofector (Lonza) with the Amaxa P3 Primary Cell 4D Nucleofector X Kit (Lonza) according to the manufacturer’s instructions. To determine subcellular localization of eGFP-CAP2, MEFs were starved for 16 h in 0.5% serum-containing medium, and then increasing the serum concentration to 10% for indicated.

### Fixation and immunohistochemistry

MEFs were plated on coverslips coated with 0.01% calf skin collagen (Sigma Aldrich) in 0.1 M acetic acid. Coverslips were fixed with 4% PFA in PBS for 10 min and afterwards washed three times with PBS for 5 min each.

For immunohistochemistry, coverslips were treated for 1 h with blocking solution and afterwards incubated over night at 4 °C in carrier solution containing the primary antibody. After three washing steps of 5 min in PBS, coverslips were incubated for 2 h at room temperature (RT) in carrier solution containing Alexa-488 conjugated secondary antibodies (1:200, Life Technologies). Afterwards, coverslips were counterstained for 10 min at RT with the intercalating dye Hoechst 33342 (Invitrogen) diluted 1:1000 in PBS. After two washing steps in PBS, coverslips were fixed on microscopic slides with Aqua-Poly/Mount (Polyscience Inc.). Images were acquired with a Leica SP5 confocal microscope using 20 × and 40 × objectives (N.A. 0.7, N.A. 1.3, respectively). Images were processed with Fiji software (ImageJ 1.51w) and analyzed by exploiting the cell counter plug-in. For determination of MRTF-A localization, cells were categorized in three categories as described before^26^: nuclear, cytoplasmic and both. For the category “both”, only cells with the same amount of MRTF-A in nucleus and cytoplasm were counted. Primary antibodies used for immunohistochemistry: mouse anti-MRTF-A (G8, Santa Cruz) and chicken anti-GFP (ab13970, Abcam.

### Treatment with LATB and JASP

Previous to treatment with LATB (Abcam, #ab1442091) or JASP (Abcam, #ab141409), pIND20-MRTF-A-GFP control and CAP2 mutant cells were activated 24 h before with 333 ng/mL doxycycline. Cells were treated for 4 h with either 25 nM LATB, 25 nM JASP or an equal volume of DMSO as control. For FRAP assay, MEFs were pretreated for 1 h with 200 nM JASP.

### Immunoblot analysis

Immunoblots were performed as described before^17^. Briefly, generation of total protein from MEFs was conducted by homogenization of cells in lysis buffer containing protease inhibitor (Complete, Roche). G/F-actin ratio of MEF cells was determined with ‘G-Actin/F-actin In Vivo Assay Biochem Kit’ (#BK037, Cytoskeleton, Inc.) according to manufacturer’s instructions. In brief, 2 × T75 flasks with 70–80% confluent cells were washed with 1 × PBS and detached from the surface using 2 mL of 0.25% Trypsin–EDTA (#25200-056, Gibco). Pelleted cells (5 min at 100×*g*, RT) were resuspended in 1.2 mL of LAS2 buffer at RT and lysed with 20 strokes in Dounce homogenizer. The lysate was incubated at 37 °C for 10 min, centrifuged at 350×*g* for 5 min at RT. G- and F-actin fractions were separated by centrifugation in Beckman SW 60 Ti rotor at 100,000×*g* for 60 min at 35 °C. F-actin fraction (pellet) was resuspended in equal volume of depolymerization buffer on ice. For SDS-PAGE, equal volumes of G- and F-actin was loaded.

Protein extracts were denatured at 95 °C in Laemmli buffer, separated by SDS-page and blotted onto a polyvinylidene difluoride membrane (Merck) by using a Wet/Tank Blotting System (Biorad). Membranes were blocked for 1 h and afterwards incubated with primary antibodies in blocking solution over night at 4 °C. As secondary antibodies, horseradish peroxidase (HRP)-conjugated antibodies (1:20,000, Thermo Fisher Scientific) were used and detected by chemiluminescence with ECL Plus Western Blot Detection System (GE Healthcare). Primary antibodies used for immunoblots: rabbit anti-CAP2 (1:1000, #15865-1-AP, Proteintech), mouse anti-actin (1:5000, #NB600-535, Novus Biologicals), rabbit anti-pan-actin (1:5000, #8456S, Cell signaling), mouse anti-alpha-tubulin (1:2000, #T9026, Sigma) and mouse anti-GAPDH (1:10,000, #MAB5718, R&D Systems).

### Quantitative PCR

Total RNA from MEFs was isolated using peqGold Trifast (VWR) according to the manufacturer’s instructions. To exclude DNA contamination, samples were treated with the TURBO DNA-free kit (Invitrogen), followed by reverse transcription using iScript cDNA synthesis kit (Bio-Rad) according to the manufacturer’s protocol. Quantitative PCR (qPCR) was performed in the STEP-One Light cycler (ABI Systems) using the iTaq SYBR Green Supermix (Bio-Rad) for detection of target genes. Three technical replicates were averaged and normalized to GAPDH in order to determine mRNA levels. Relative changes were calculated using the ΔΔCt method. Primer sequences: SRF (forward (f): TTCCCGTCCGAGGAAACAT, reverse (r) GGCTCTTTTGACCCAGACCAT), Vinculin (f: AGCCCAGATGCTTCAGTCAGA, r: GGTCAGATGTGCCAGAAAGGA), c-Fos (f: TTCCTACTACCATTCCCCAGCC, r: GATCTGCGCAAAAGTCCTGTG, GAPDH (f: CCCTTCATTGACCTCAACTA, r: CCAAAGTTGTCATGGATGAC), ACTA2 (f: GGCATCCACGAAACCACCTAT, r: CTGTGATCTCCTTCTGCATCCT), EGR2 (f: TGCTAGCCCTTTCCGTTGA, r: TCTTTTCCGCTGTCCTCGAT), CYR61 (f: AATCGCAATTGGAAAAGGCA, r: TGAAAAGAACTCGCGGTTCG).

### Cell viability

Cell proliferation was evaluated by using electrical impedance monitoring, through the xCELLigence Real-Time Cell Analysis (RTCA; Roche Diagnostics) system.

When cells proliferate, the current flow between the microelectrodes, placed in the bottom surface of each well of a 96-well plate, is impeded. The impedance of this electron flow is reported as arbitrary cell index-values. For this assay, CTR and KO MEF cells were seeded in a 96-well plate (6000 cells/well) and treated or not with erastin (0.7 µM; Calbiochem).

Metabolic activity as an indicator for cell viability was assessed through the 3-[4,5-dimethylthiazole-2-yl]-2,5-diphenyltetrazolium bromide (MTT) assay. In viable and metabolically active cells, MTT is reduced to a purple formazan. CTR and KO MEF cells have been seeded in 96-well plates (2000 cells/well) and treated with erastin (0.7 µM) for 8 h. Subsequently, MTT (0.5 mg/mL; Merck) was added for 1 h at 37 °C for the purple formazan production. Absorbance was measured at 570–630 nm with FLUOstar (BMG Labtech).

### Mitochondrial morphology

CTR and KO MEF cells were seeded in 8-well ibidi slides (Ibidi GmbH) at a density of 7000 cells per well, stained with MitoTracker Deep Red FM (200 nM for 30 min at 37 °C; Invitrogen) and fixed with 4% paraformaldehyde for 20 min at room temperature. Images were acquired using a Leica DM6000 epi-fluorescence microscope (63 × objective), by using an excitation wavelength of 620 nm and detecting emission using a 670 nm filter (red). Mitochondrial shape was classified as described before^29^: (i) category I comprises cells with healthy, elongated and equally distributed mitochondria, which are organized in a tubular network; (ii) category II comprises cells with partially fragmented mitochondria, which are still distributed throughout the cytosol, (iii) category III comprises cells with completely fragmented mitochondria, accumulating around the nucleus. At least 500 cells were counted by an experimenter blinded to the genotype.

### Mitochondrial superoxide formation

CTR and KO MEF cells, seeded in 24-well plate (10,000 cells/well), have been treated or not with erastin (0.7 µM) for 8 h. Subsequently, stained with MitoSOX Red (1.25 µM; Invitrogen) for 30 min at 37 °C, cells were harvested for FACS analysis (excitation 488 nm, emission 690/50 nm; Guava easyCyte Flow Cytometer, Merck). Increased red fluorescence has been correlated with the formation of mitochondrial reactive oxygen species (ROS). Data were collected from at least 5000 cells and four replicates per condition.

### Lipid peroxidation

CTR and KO MEF cells, seeded in 24-well plate (10,000 cells/well), have been treated or not with erastin (0.7 µM) for 8 h. Subsequently, stained with BODIPY 581/591 C11 (2 µM; Thermo Fisher Scientific) for 1 h at 37 °C, cells were harvested for subsequent FACS analysis (excitation 488 nm, emission 525/30 nm and 585/50 nm). The shift from red to green fluorescence was used to analyze lipid peroxidation. Data were collected from at least 5000 cells and four replicates per condition.

### Mitochondrial membrane potential

Mitochondrial membrane potential was analyzed by using the MitoPT TMRE Kit (ImmunoChemistry Technologies). CTR and KO MEF cells were seeded in 24-well plate (10,000 cells/well), treated or not with erastin (0.7 µM) for 16 h. Cells were stained with TMRE (0.2 µM) for 30 min at 37 °C and harvested for subsequent FACS analysis (excitation 488 nm, emission 690/50 nm). A decrease of TMRE fluorescence was representative of a loss of mitochondrial membrane potential. Data were collected from at least 5000 cells and four replicates per condition.

### Statistical analysis

**Figure 1:**
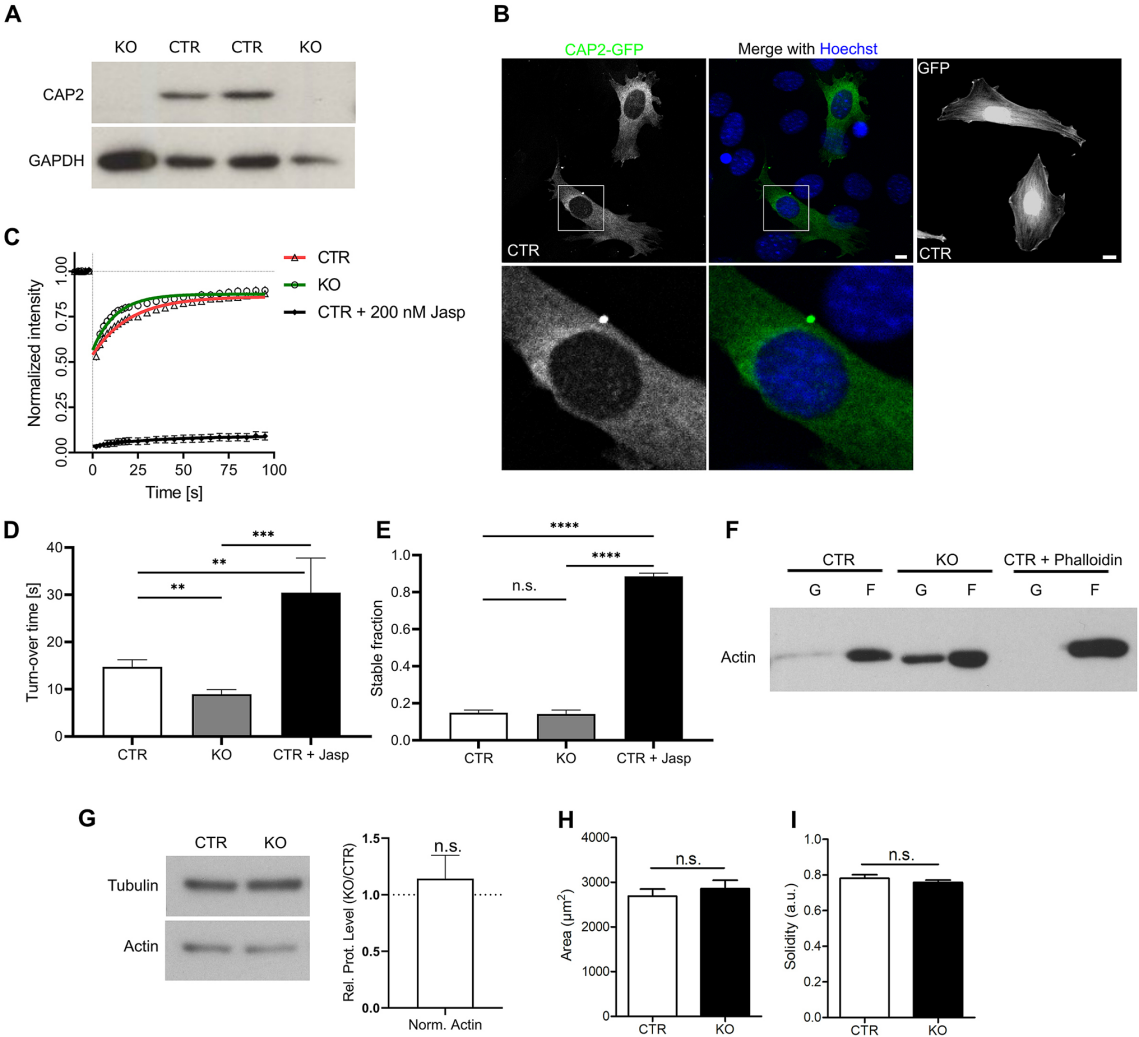
CAP2 inactivation increased G-actin levels in MEFs. (**A**) Immunoblots showing CAP2 expression in two CTR MEF lines and CAP2 inactivation in two KO MEF lines. GAPDH was used as loading control. (**B**) Representative micrographs showing localization of GFP-tagged CAP2 (green) in CTR MEFs (left panel). Merge micrograph (middle panel) includes counterstaining with DNA dye Hoechst (blue). Box indicates area shown at higher magnification. Representative micrograph of GFP-transfected CTR MEF (right panel). (**C**) Fluorescence recovery curve after photobleaching in GFP-actin transfected CTR (red) and KO MEFs (green) as well as in CTR MEFs treated with 200 nM jasplakinolide (JASP; black). (**D**) Half-recovery time of actin turnover and (**E**) stable actin fraction in CTR and KO MEFs as well as in JASP-treated CTR MFEs. (**F**) Representative immunoblots showing actin in soluble (**G**) and insoluble (**F**) protein fractions. (**G**) Immunoblot showing total actin levels in CTR and KO MEFs (left). Quantification of relative protein levels from 5 biological replicates (see Fig. S2). Signal intensity of actin was first normalized to tubulin, and then ratio of KO vs CTR was calculated (right). (**H**) Area quantification of CTR and KO MEFs. (**I**) Solidity index of CTR and KO MEFs. Scale bar in (**B**): 10 µm. *P < 0.05, **P < 0.01, ***P < 0.001, *ns* not significant.

**Figure 2:**
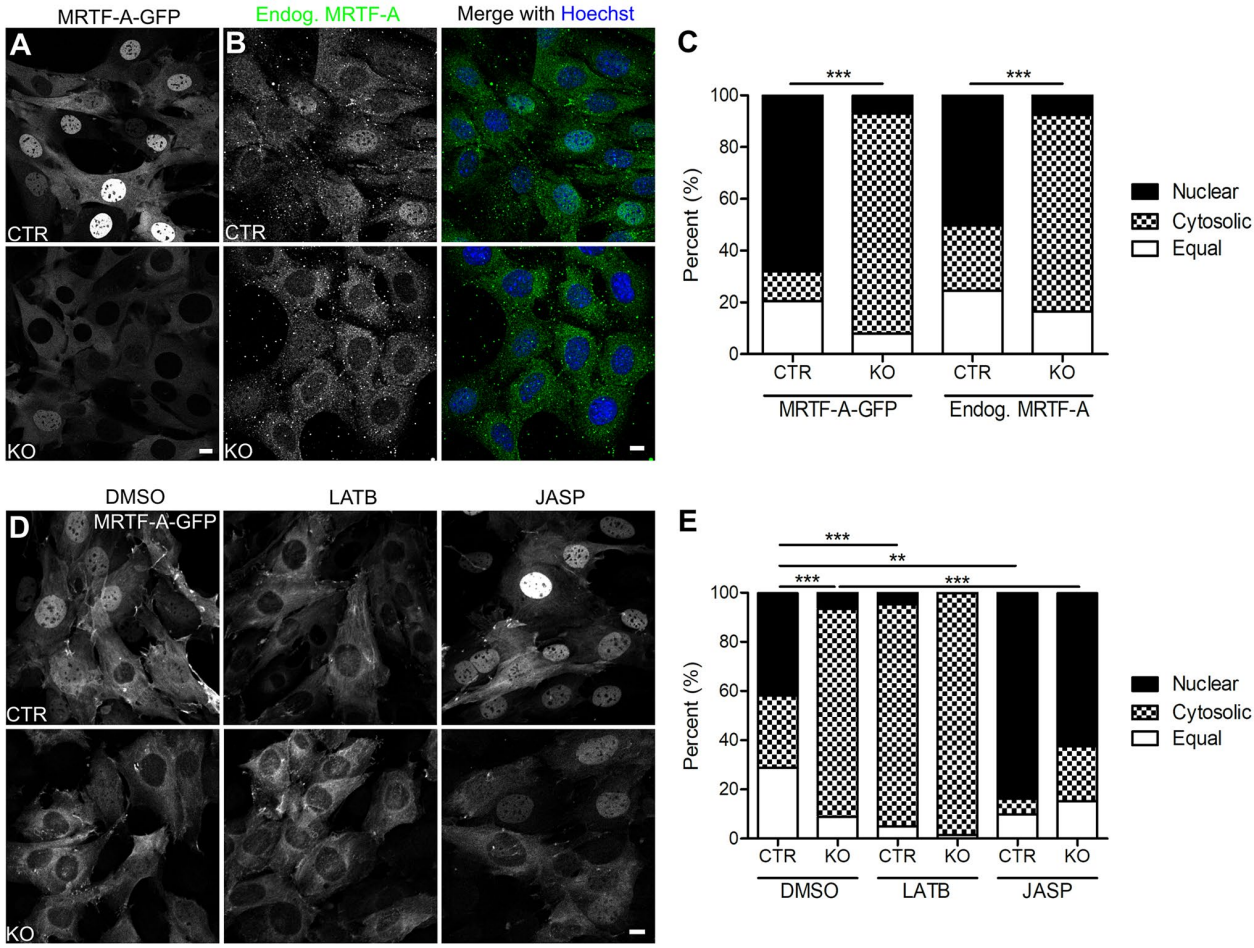
CAP2 controls MRTF-A localization in an actin-dependent manner. (**A**) Representative micrographs of CTR and KO MEFs that stably expressed GFP-tagged MRTF-A (MRTF-A-GFP). (**B**) Representative micrographs of CTR and KO MEFs stained with an antibody against MRTF-A (green). MEFs were counterstained with the DNA-dye Hoechst (blue). (**C**) Categorization of MEFs according to the localization of MRTF-A-GFP or endogenous MRTF-A, i.e. fractions with mainly nuclear or cytosolic MRTF-A-GFP and fraction with equal levels in both compartments. (**D**) Representative micrographs of MRTF-A-GFP-expressing CTR and KO MEFs upon treatment with either DMSO, latrunculin B (LATB) or jasplakinolide (JASP). (**E**) Categorization of MEFs according to the localization of MRTF-A-GFP upon treatment with either DMSO, LATB or JASP. Scale bars (in µm): 10 (**A**,**B**,**D**). *P < 0.05, **P < 0.01, ***P < 0.001, *ns* not significant.

**Figure 3:**
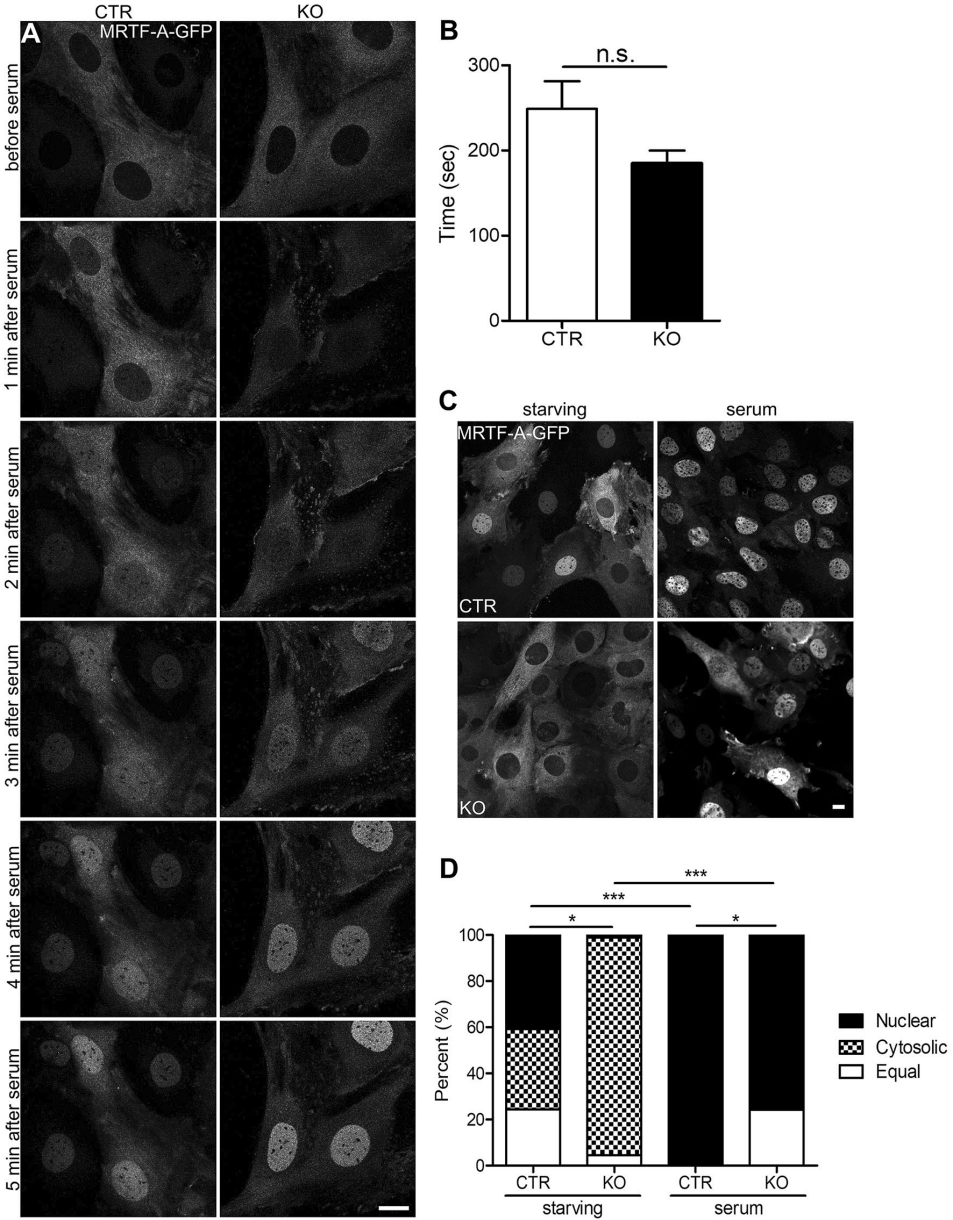
CAP2 was dispensable for serum induced nuclear MRTF-A translocation in MEFs. (**A**) Image sequence of MRTF-A-GFP-expressing MEFs before and during serum stimulation. (**B**) Latency of nuclear MRTF-A translocation. (**C**) Representative micrographs of MRTF-A-GFP-expressing CTR and KO MEFs during serum starvation and upon serum stimulation. (**D**) Categorization of MEFs according to the localization of MRTF-A-GFP or endogenous MRTF-A during serum starvation and upon serum stimulation. Scale bars (in µm): 10 (**A**,**C**). *P < 0.05, **P < 0.01, ***P < 0.001, *ns* not significant.

**Figure 4:**
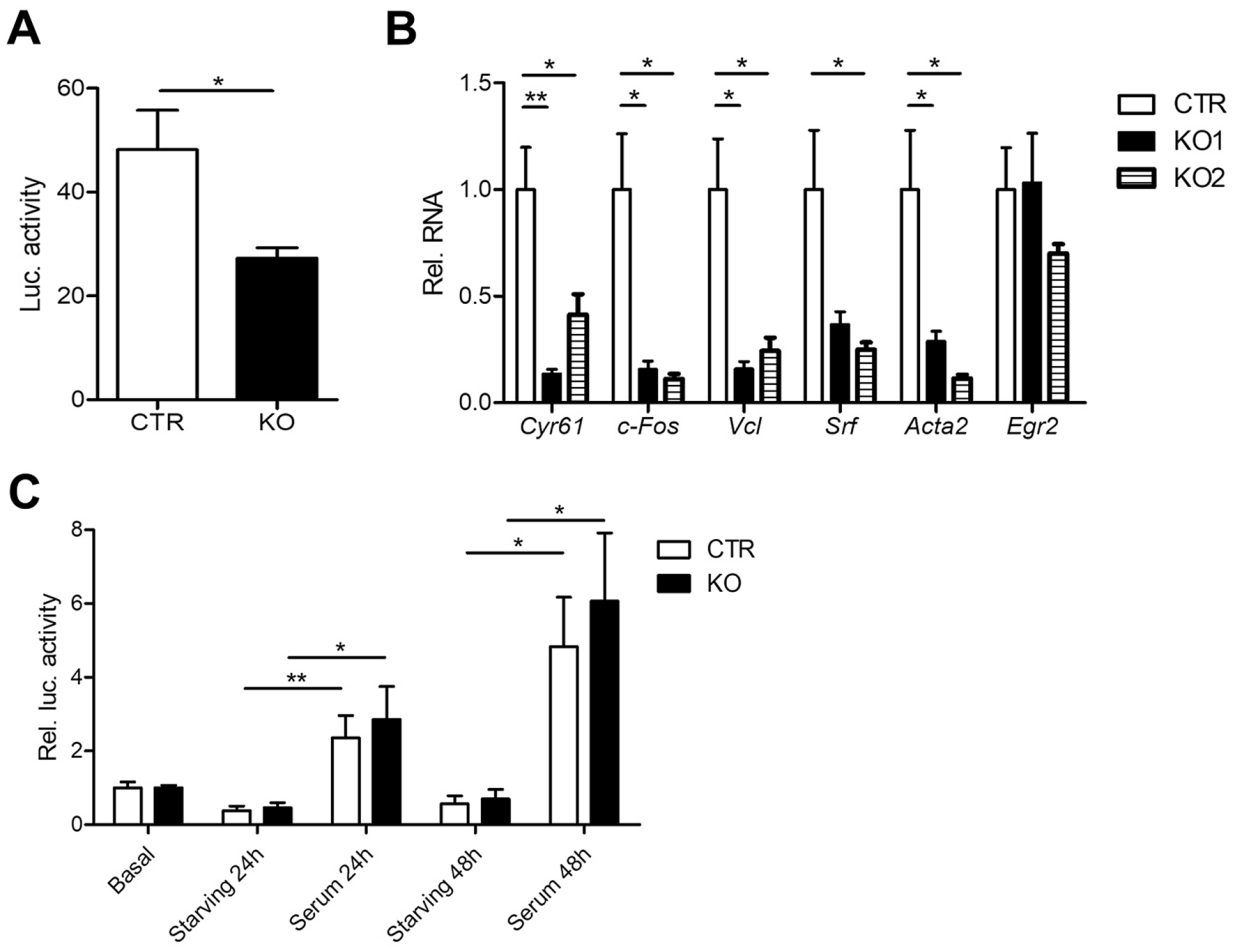
CAP2 inactivation reduced SRF activity in MEFs. (**A**) Firefly luciferase activity in CTR and KO MEFs that stably expressed a SRF reporter in which firefly luciferase expression was under control of SRF activity. (**B**) mRNA levels of selected SRF target genes in two KO MEF lines as determined by qPCR. (**C**) Luciferase expression in CTR and KO MEFs under basal conditions, during starving and upon serum stimulation for either 24 or 48 h. Values are normalized to basal levels. *P < 0.05, **P < 0.01, ***P < 0.001, *ns* not significant.

Statistical analysis was performed using the Prism statistical analysis package (GraphPad Software). Data are expressed as mean ± standard error (SE) for all main figures and mean ± standard deviation (SD) for the supplementary figure. All experiments have been conducted in at least three independent experiments. For supplementary figure eight replicates per condition were used if not specified. Data sets followed a normal distribution and differences between groups were evaluated by either one- or two-way ANOVA followed by post-hoc Dunns’ or Scheffé’s test (Fig. 4C, Fig. S3A), Chi-square test (Figs. 2C,E, 3D) or Student’s *t* test (Figs. 1D,E,G–I,K, 3B, 4A,B) and considered significant at p < 0.05. All experiments were conducted by experimenters blind to the genotype.

## Results

### CAP2 controls actin turnover and equilibrium in mouse fibroblasts

To study the cellular function of CAP2 in mammalian cells, we generated immortalized mouse embryonic fibroblasts (MEFs) from two CAP2^−/−^ mice (termed KO) and two CAP2^+/+^ control littermates (CTR) at embryonic day (E) 12.5. Immunoblots confirmed absence of CAP2 from both KO MEF lines (Fig. 1A). Due to the lack of specific antibodies suitable for immunocytochemistry, we examined subcellular CAP2 localization in MEFs by expressing a green fluorescent protein (GFP)-tagged CAP2 construct. GFP-CAP2 was homogenously distributed within the cytosol and largely absent from the nucleus (Fig. 1B).

Since CAP2 has been implicated in actin regulation^12^, we first tested whether KO MEFs displayed actin defects. We therefore performed fluorescence recovery after photobleaching (FRAP) experiments in GFP-actin-expressing CTR and KO MEFs to determine actin turnover (Fig. 1C, Fig. S1; Movies S1–S2). In CTR MEFs, GFP-actin rapidly recovered with a mean half-recovery time (t_1/2_) of 14.73 ± 1.51 s (n = 20 cells from 3 independent experiments; Fig. 1D). Compared to CTR MEFs, fluorescence recovery was faster in KO MEFs (8.95 ± 0.95 s, n = 17/3, P < 0.01). Instead, the stable actin fraction that did not recover within 95 s was not different between CTR and KO MEFs (Fig. 1E; CTR: 0.15 ± 0.02, KO: 0.14 ± 0.02 n = 20/3 and 17/3, respectively; P = 0.819). Hence, inactivation of CAP2 increased actin turnover, but did not alter the stable actin fraction. Treatment of CTR MEFs with jasplakinolide (JASP), a potent inducer of actin polymerization, drastically increased both t_1/2_ (30.44 ± 7.34 s, n = 10/2, P < 0.001) as well as stable actin fraction (0.89 ± 0.02, n = 10/2, P < 0.001), thereby confirming suitability of the approach for determining actin turnover (Fig. 1D,E, Fig. S1, Movie S3). Next, we tested whether CAP2 was relevant for the equilibrium between G-actin and F-actin. To do so, we lysed MEFs in a buffer optimized to stabilize and maintain G- and F-forms of cellular actin, similar to previous studies^30^. This approach revealed an increase in the G-actin fraction in KO MFEs, suggesting a shift in the equilibrium towards G-actin (Fig. 1F, Fig. S2). The G-actin increase in KO MEFs was not due to changes in total actin levels (Fig. 1G, Fig. S2). Together, CAP2 inactivation in MEFs caused faster actin turnover and a shift in the actin equilibrium towards G-actin, while it did alter total actin levels.

Next, we tested whether these actin changes were associated with cellular defects. We found that KO MEFs normally adhered to cell culture dishes and did not differ from CTR MEFs in size or solidity index that we calculated to assess cellular morphology (Fig. 1H,I: size (in µm^2^): CTR: 2689.45 ± 154.77, KO: 2856.97 ± 184.21, n ≥ 25/3, P = 0.500; solidity index (arbitrary units): CTR: 0.78 ± 0.02, KO: 0.76 ± 0.01, n ≥ 25/3 cells, P = 0.316). Further, real-time impedance measurements revealed no differences between CTR and KO MEFs in cell proliferation (Fig. S3A). Since previous studies revealed defects in mitochondrial morphology and function upon inactivation of actin regulators and putative CAP2 interaction partners including cofilin1 or inverted formin 2 (INF2^31^, Rehklau et al. 2017^8–10,12,32^, we tested whether CAP2 inactivation affected mitochondrial morphology or function. We found that CAP2 inactivation did not affect metabolic activity, mitochondrial morphology, mitochondrial ROS production, lipid peroxidation or mitochondrial membrane potential (Fig. S3B–G). Furthermore, oxidative stress induced by erastin treatment similarly changed metabolic and mitochondrial parameters in CTR and KO MEFs^33,34^. Together, morphology, proliferation, metabolic activity, mitochondrial function and response to oxidative stress were unchanged in KO MEFs. Hence, actin defects in KO MEFs were not associated with any obvious cellular defects.

### CAP2 inactivation alters subcellular distribution of MRTF-A in mouse fibroblasts

MRTF-A is a transcriptional coactivator that shuttles between the cytosol and the nucleus. This shuttling depends on G-actin, because G-actin binding is necessary for nuclear MRTF-A export and interferes with accessibility of its nuclear localization sequence^35,36^. Hence, the shift towards G-actin in KO MEFs might be associated with altered subcellular MRTF-A distribution. To test this, we generated CTR and KO MEF lines that stably expressed GFP-tagged MRTF-A (MRTF-A-GFP) and grouped MEFs into three categories: (1) MEFs with mainly nuclear MRTF-A-GFP (nuclear), (2) MEFs with mainly cytosolic MRTF-A-GFP (cytosolic) and (3) MEFs with equal MRTF-A-GFP levels in both compartments (equal), similar to previous studies^26^. In KO MEFs, MRTF-A-GFP distribution was different from CTR MEFs, as indicated by an almost sevenfold increase in the cytosolic MRTF-A-GFP fraction and a sevenfold decrease in the nuclear MRTF-A-GFP fraction (Fig. 2A,C; (in %) CTR: nuclear: 68.04 ± 3.74, cytosolic: 11.51 ± 2.58, equal: 20.45 ± 2.22, n = 15/3, KO: nuclear: 9.00 ± 2.09, cytosolic: 85.22 ± 2.61, equal: 5.78 ± 1.95, n = 15/3, P < 0.001). In an independent experiment, we determined localization of endogenous MRTF-A by immunocytochemistry and found very similar differences between CTR and KO MEFs (Fig. 2B,C; (in %) CTR: nuclear: 50.24 ± 6.87, cytosolic: 25.23 ± 3.60, equal: 24.53 ± 4.77, n = 6/3, KO: nuclear: 7.50 ± 2.12, cytosolic: 75.97 ± 4.38, equal: 16.53 ± 3.24, n = 6/3, P < 0.001), thereby demonstrating that our GFP-tagged construct faithfully reflected MRTF-A localization and that our stably transfected MEF lines were valuable tools to study the mechanisms that control MRTF-A localization. Together, increased G-actin levels in KO MEFs were associated with altered MRTF-A localization, and we therefore hypothesized that CAP2 controls MRTF-A localization in an actin-dependent manner.

### F-actin stabilization restored MRTF-A localization in CAP2-deficient MEFs

To test this hypothesis, we exploited MRTF-A-GFP expressing MEF lines to determine MRTF-A localization upon pharmacological manipulation of the actin cytoskeleton. Latrunculin B (LATB) reportedly increased G-actin levels and promoted interaction of G-actin with MRTF-A^37^. Hence, LATB treatment should increase cytosolic MRTF-A localization^20^. Indeed, when compared to dimethyl sulfoxide (DMSO)-treated CTR MEFs, LATB increased the CTR MEF fraction with cytosolic MRTF-A-GFP localization and it substantially decreased the fraction with nuclear localization (Fig. 2D,E; DMSO: nuclear: 41.76 ± 2.68, cytosolic: 29.40 ± 2.64, equal: 28.85 ± 2.25, LATB: nuclear: 4.95 ± 2.36, cytosolic: 90.20 ± 3.43, equal: 4.85 ± 1.86, n = 9/3, P < 0.01). In contrast, LATB failed in changing the subcellular MRTF-A-GFP distribution in KO MEFs (DMSO: nuclear: 6.74 ± 1.43, cytosolic: 84.46 ± 2.77, equal: 8.81 ± 1.87, LATB: nuclear: 0.28 ± 0.26, cytosolic: 98.35 ± 0.64, equal: 1.38 ± 0.66, n = 9/3, P = 0.158). Notably, subcellular MRTF-A distribution was not different between CTR and KO MEFs upon LATB treatment (P = 0.225). Hence, LATB caused a subcellular MRTF-A distribution in CTR MEFs that was similar to that in KO MEFs.

Apart from LATB, we tested JASP that stabilizes F-actin, reduces G-actin levels and that reportedly induced nuclear import of MRTF-A^20,38^. As expected, JASP doubled the fraction of CTR MEFs with nuclear MRTF-A-GFP localization and reduced the fraction with cytosolic localization (Fig. 2D,E; JASP: nuclear: 83.69 ± 2.61, cytosolic: 6.41 ± 1.05, equal: 9.90 ± 2.16, n = 9/3, P < 0.01). Similarly, JASP changed the subcellular MRTF-A-GFP distribution in KO MEF as indicated by a ninefold increased fraction with nuclear MRTF-A-GFP localization concomitant with a fourfold decreased cytosolic fraction (JASP: nuclear: 62.21 ± 6.32, cytosolic: 22.28 ± 5.95, equal: 15.50 ± 2.19, n = 9/3, P < 0.01). Notably, the subcellular MRTF-A distribution did not differ between CTR and KO MEFs upon JASP treatment (P = 0.237). Hence, pharmacologically induced reduction of G-actin levels restored MRTF-A localization in KO MEFs. These data suggested that CAP2 controlled MRTF-A localization in an actin-dependent manner.

### CAP2 is dispensable for serum induced nuclear MRTF-A translocation

Serum stimulation reportedly induced nuclear MRTF-A translocation in fibroblasts^21,23,39^. By exploiting MRTF-A-GFP expressing MEF lines, we next tested whether CAP2 was relevant for serum-induced nuclear import of MRTF-A. First, we starved MEFs in 0.3% fetal calf serum (FCS) for 48 h and, thereafter, determined nuclear translocation during the first six min of stimulation with 20% FCS by live-cell imaging. We restricted this analysis to CTR and KO MEFs of the ‘cytosolic MRTF-A fraction’ to avoid any false interpretation due to different MRTF-A localization before stimulation. As obvious from the movies and image sequences (Fig. 3A, Movies S4–S5), MRTF-A rapidly translocated into the nucleus in CTR and KO MEFs upon serum stimulation. Quantification of the latency of nuclear translocation revealed no difference between both groups (Fig. 3B; (in s) CTR: 248.75 ± 31.25, KO: 185.00 ± 14.18, n = 12/3, P = 0.089). Next, we determined subcellular MRTF-A localization in all CTR and KO MEFs both upon 48 h of serum starvation and upon 10 min of serum stimulation. Compared to basal conditions (Fig. 2C), the CTR MEF fraction with nuclear MRTF-A localization was reduced by 40%, and the fraction with cytosolic MRTF-A was increased threefold upon starvation (Fig. 3C,D; nuclear: 40.90 ± 3.78, cytosolic: 34.73 ± 5.02, equal: 24.38 ± 3.63, n = 25/3, P < 0.001). As expected, serum stimulation induced a nuclear translocation of all MRTF-A in CTR MEFs, and we did not note CTR MEFs with predominantly cytosolic MRTF-A or equal localization in cytosol and nucleus (Fig. 3C,D; n = 9/3, P < 0.001). In contrast to CTR MEFs, serum starvation did not alter MRTF-A localization in KO MEFs (nuclear: 1.67 ± 1.07, cytosolic: 92.34 ± 2.39, equal: 6.00 ± 2.29, n = 15/3, P < 0.0543), while serum stimulation induced nuclear MRTF-A translocation in the majority of KO MEFs. However, unlike in FCS-stimulated CTR MEFs, we noted a fraction of 25% KO MEFs with equal localization of MRTF-A in cytosol and nucleus upon FCS stimulation (nuclear: 75.02 ± 3.79, equal: 24.98 ± 3.79, n = 9/3, P < 0.001). Upon serum stimulation, subcellular MRTF-A distribution was still different between CTR and KO MEFs (P < 0.05). In order to determine whether starvation and subsequent serum stimulation affects subcellular localization of CAP2, we overexpressed GFP-CAP2 in CTR MEF cells. Unlike MRTF-A, the cytoplasmic localization of GFP-CAP2 was not changed under those conditions (Fig. S4). Together, serum stimulation induced nuclear MRTF-A translocation in KO MEFs with a latency similar to CTR MEFs. However, different from CTR MEFs, in a quarter of serum-stimulated KO MEFs MRTF-A was still present in the cytosol.

### CAP2 inactivation reduces SRF activity in mouse fibroblasts

So far, we showed that CAP2 (i) controls MRFT-A localization in MEFs and (ii) was largely dispensable for serum-induced nuclear MRTF-A translocation. Next, we tested whether CAP2 was relevant for MRFT-A-dependent activation of SRF. To do so, we generated CTR and KO MEF lines that stably expressed a SRF reporter in which expression of firefly luciferase was controlled by three minimal c-Fos promoter sequences including serum response elements, but lacking TCF binding sites^26^. Luciferase activity was strongly reduced in KO MEFs and reached only half of that in CTR MEFs, thereby suggesting reduced SRF activity in KO MEFs (Fig. 4A; (arbitrary units) CTR: 48.19 ± 6.94, KO: 27.28 ± 1.81, n = 6, P < 0.05). Indeed, qPCR experiments revealed a reduction in mRNA levels for several established SRF downstream target genes including those encoding for c-Fos (*c-Fos*), vinculin (*Vcl*), smooth muscle α-actin (*Acta2*), Cyr61 (*Cyr61*) or SRF (*Srf*) itself (Fig. 4B). In these experiments, we found similar changes in both KO MEF lines generated (KO 1: *Cyr61*: 0.16 ± 0.02, P < 0.01; *c-Fos*: 0.16 ± 0.03, P < 0.05; *Acta2*: 0.29 ± 0.05, P < 0.05; *Vcl*: 0.20 ± 0.05, P < 0.05; *Srf*: 0.48 ± 0.07, P = 0.073, n = 9; KO 2: *Cyr61*: 0.48 ± 0.11, P < 0.05; *Fos*: 0.11 ± 0.02, P < 0.05; *Acta2*: 0.11 ± 0.02, P < 0.05; *Vcl*: 0.24 ± 0.06, P < 0.05; *Srf*: 0.29 ± 0.04, P < 0.05, n = 9). Notably, mRNA levels of *Egr2*, a SRF downstream target that is controlled by the TCF, but not by the MRTF-A pathway, was unchanged in both KO MEF lines (KO 1: 1.03 ± 0.20, n = 9, P = 0.923; KO 2: 0.70 ± 0.03, n = 9, P = 0.319). Together, altered MRTF-A localization in KO MEFs was associated with reduced SRF activity.

Next, we wanted to know whether serum-induced SRF activity depends on CAP2. To test this, we starved CTR and KO MEFs expressing the SRF reporter for 20 h before stimulation with 20% FCS for either 24 or 48 h. As expected, serum stimulation for either 24 or 48 h induced SRF activity in CTR MEFs (Fig. 4C; 24 h: 2.36 ± 0.55, n = 6, P < 0.01; 48 h: 4.83 ± 1.22, n = 6, P < 0.05). Very similar to CTR MEFs, serum stimulation induced SRF activity in KO MEFs (24 h: 2.86 ± 0.81, n = 6, P < 0.05; 48 h: 6.06 ± 1.69, n = 6, P < 0.05), and serum induced SRF activity was not different between groups (24 h: P = 0.652; 48 h: P = 0.601). Together, basal SRF activity was reduced in KO MEFs, but serum stimulation equally induced SRF activity in CTR and KO MEFs.

## Discussion

The present study aimed at deciphering the CAP2-dependent mechanism relevant for the control of the transcription factor SRF. We chose mouse embryonic fibroblasts (MEFs) as a cellular model system for our study, because in these cells CAP2 is expressed at substantial levels and SRF-dependent gene regulation and upstream regulatory mechanisms have been intensively studied^21^. We found a shift in the actin equilibrium towards G-actin in CAP2 mutant MEFs, which was associated with a reduction in nuclear MRTF-A, reduced SRF activity and decreased expression of established MRTF-SRF target genes. While drug-induced increase in G-actin levels altered MRTF-A localization in control MEFs, it did not affect MRTF-A localization in mutant MEFs, which was normalized upon drug-induced decrease in G-actin levels. These data let us propose a model in which CAP2 controls SRF-dependent gene expression via regulating G-actin levels and nuclear MRTF-A localization.

This model is in good agreement with recent studies that identified important functions for CAPs in controlling F-actin dynamics^5–12^. Specifically, these studies showed that CAPs can (i) facilitate F-actin severing and actin subunit dissociation in synergy with ADF/cofilin and twinfilin^5–9,11^, (ii) catalyze ATP-for-ADP exchange on G-actin that is relevant for F-actin assembly^6,40–42^, and (iii) inhibit activity of INF2 that promotes F-actin assembly^10^. Hence, by regulating various aspects of F-actin assembly and disassembly, CAPs control G-actin levels and, hence, interaction of G-actin with MRTF-A. Our finding of increased G-actin levels and reduced nuclear MRTF-A localization in CAP2 mutant MEFs, together with normalization of MRTF-A localization upon treatment with the F-actin stabilizing drug excluded that CAP2 acts primarily as a F-actin disassembly factor in MEFs.

We found reduced SRF activity in reporter assays as well as reduced expression of SRF target genes in CAP2 mutant MEFs. Supportively, reduced expression of MRTF-SRF targets in CAP2 mutant MEFs have been noted recently by others^19^. Although SRF activity has not been systematically analyzed in skeletal muscle from CAP2 mutant mice, decreased mRNA levels of established MRTF-SRF targets such as *Acta1*, *Acta2* and *Actc1* point towards reduced SRF activity in skeletal muscles from CAP2 mutant embryos, too^17^, in which SRF dysregulation may contribute to retarded skeletal muscle development. Indeed, a crucial function for the MRTF-SRF pathway during late embryonic skeletal muscle development is evident from gene-targeted mice^43,44^. Opposite to our findings in CAP2 mutant MEFs and to reduced expression of MRTF-SRF target genes in skeletal muscles from CAP2 mutant embryos^17^, a recent study reported upregulation of several MRTF-SRF target genes including *Acta1* and *Acta2* in heart tissue and isolated cardiomyocytes from CAP2 mutant mice, which was associated with increased nuclear MRTF levels. Interestingly, heart defects in CAP2 mutant mice including dilated cardiomyopathy and impaired cardiac conductance were partially restored upon pharmacological inhibition of MRTF-SRF activity, demonstrating that CAP2-dependent regulation of the MRTF-SRF pathway is physiologically relevant and that its dysregulation due to CAP2 inactivation contributes to pathological conditions^19^. The opposite effects of CAP2 inactivation on MRTF-SRF activity suggest different CAP2 activities towards F-actin dynamics in MEFs versus cardiomyocytes. Unlike in MEFs and presumably in skeletal muscle, CAP2 may primarily act as a F-actin disassembly factor in cardiomyocytes.

By chromatin immunoprecipitation combined with deep sequencing, previous studies convincingly demonstrated that serum-induced, SRF-mediated transcriptional response largely depends on the MRTF-SRF pathway^21^. In line with these data, we showed efficient MRTF-A translocation into the nucleus as well as elevated SRF activity upon serum stimulation in control MEFs. Interestingly, serum-stimulated nuclear MRTF-A translocation as well as SRF activation was similar to controls in CAP2 mutant MEFs. Hence, while we found a role for CAP2 in MRTF-A localization and SRF activity under basal conditions, in unstimulated MEFs, CAP2 was dispensable for serum stimulation of the MRTF-SRF pathway.

Via acting on transmembrane receptors including G-protein-coupled receptors, receptor tyrosine kinases or serine-threonine receptor kinases, extracellular signals are translated into intracellular signaling cascades that include Rho family small guanosine triphosphatases (GTPases)^45–47^. Effectors of Rho GTPases include, among others, formins, Wiskott–Aldrich syndrome protein (WASP), WASP-family verprolin homologues (WAVEs) and actin-related protein 2/3 (ARP2/3) complex, which orchestrate actin polymerization^48^. Hence, Rho GTPase signaling promotes incorporation of G-actin into filaments that releases MRTF from G-actin complexes and stimulates MRTF-SRF-dependent gene expression^20^. Rho GTPase signaling further shifts the F/G-actin equilibrium towards F-actin via activation of Rho-associated kinases (ROCKs) that in turn inhibits actin depolymerizing proteins of the ADF/cofilin family^49^. ADF/cofilin cooperates with CAPs in actin dynamics^5,6,8,9,12,50^, and inhibition of ADF/cofilin activity upon serum simulation may explain why CAP2 was dispensable for serum-induced stimulation of the MRTF-SRF pathway. In summary, our data revealed that CAP2 controls the subcellular localization of MRTF and thereby SRF activity in unstimulated MEFs, while CAP2 was dispensable for serum-induced nuclear MRTF translocation and MRTF-SRF stimulation.

## Supplementary Information

Supplementary Information.

Supplementary Movie S1.

Supplementary Movie S2.

Supplementary Movie S3.

Supplementary Movie S4.

Supplementary Movie S5.

## References

[1] Balcer HI, Goodman AL, Rodal AA, Smith E, Kugler J, Heuser JE, Goode BL (2003). Coordinated regulation of actin filament turnover by a high-molecular-weight Srv2/CAP complex, cofilin, profilin, and Aip1. Curr. Biol..

[2] Bertling E, Hotulainen P, Mattila PK, Matilainen T, Salminen M, Lappalainen P (2004). Cyclase-associated protein 1 (CAP1) promotes cofilin-induced actin dynamics in mammalian nonmuscle cells. Mol. Biol. Cell.

[3] Freeman NL, Field J (2000). Mammalian homolog of the yeast cyclase associated protein, CAP/Srv2p, regulates actin filament assembly. Cell Motil. Cytoskeleton.

[4] Hubberstey AV, Mottillo EP (2002). Cyclase-associated proteins: CAPacity for linking signal transduction and actin polymerization. FASEB J..

[5] Chaudhry F, Little K, Talarico L, Quintero-Monzon O, Goode BL (2010). A central role for the WH2 domain of Srv2/CAP in recharging actin monomers to drive actin turnover in vitro and in vivo. Cytoskeleton (Hoboken, NJ).

[6] Jansen S, Collins A, Golden L, Sokolova O, Goode BL (2014). Structure and mechanism of mouse cyclase-associated protein (CAP1) in regulating actin dynamics. J. Biol. Chem..

[7] Johnston AB, Collins A, Goode BL (2015). High-speed depolymerization at actin filament ends jointly catalysed by Twinfilin and Srv2/CAP. Nat. Cell Biol..

[8] Kotila T, Kogan K, Enkavi G, Guo S, Vattulainen I, Goode BL, Lappalainen P (2018). Structural basis of actin monomer re-charging by cyclase-associated protein. Nat. Commun..

[9] Kotila T (2019). Mechanism of synergistic actin filament pointed end depolymerization by cyclase-associated protein and cofilin. Nat. Commun..

[10] Mu A, Fung TS, Kettenbach AN, Chakrabarti R, Higgs HN (2019). A complex containing lysine-acetylated actin inhibits the formin INF2. Nat. Cell Biol..

[11] Shekhar S, Chung J, Kondev J, Gelles J, Goode BL (2019). Synergy between cyclase-associated protein and Cofilin accelerates actin filament depolymerization by two orders of magnitude. Nat. Commun..

[12] Rust MB, Khudayberdiev S, Pelucchi S, Marcello E (2020). CAPt’n of actin dynamics: Recent advances in the molecular, developmental and physiological functions of cyclase-associated protein (CAP). Front. Cell Dev. Biol..

[13] Jang HD (2019). Cyclase-associated protein 1 is a binding partner of proprotein convertase subtilisin/kexin type-9 and is required for the degradation of low-density lipoprotein receptors by proprotein convertase subtilisin/kexin type-9. Eur. Heart J..

[14] Field J (2015). CAP2 in cardiac conduction, sudden cardiac death and eye development. Sci. Rep..

[15] Peche VS (2012). Ablation of cyclase-associated protein 2 (CAP2) leads to cardiomyopathy. Cell. Mol. Life Sci..

[16] Stockigt F, Peche VS, Linhart M, Nickenig G, Noegel AA, Schrickel JW (2016). Deficiency of cyclase-associated protein 2 promotes arrhythmias associated with connexin43 maldistribution and fibrosis. Arch. Med. Sci..

[17] Kepser LJ, Damar F, De Cicco T, Chaponnier C, Proszynski TJ, Pagenstecher A, Rust MB (2019). CAP2 deficiency delays myofibril actin cytoskeleton differentiation and disturbs skeletal muscle architecture and function. Proc. Natl. Acad. Sci. U.S.A..

[18] Peche V (2007). CAP2, cyclase-associated protein 2, is a dual compartment protein. Cell. Mol. Life Sci..

[19] Xiong Y (2019). Targeting MRTF/SRF in CAP2-dependent dilated cardiomyopathy delays disease onset. JCI Insight.

[20] Olson EN, Nordheim A (2010). Linking actin dynamics and gene transcription to drive cellular motile functions. Nat. Rev. Mol. Cell Biol..

[21] Esnault C, Stewart A, Gualdrini F, East P, Horswell S, Matthews N, Treisman R (2014). Rho-actin signaling to the MRTF coactivators dominates the immediate transcriptional response to serum in fibroblasts. Genes Dev..

[22] Gualdrini F, Esnault C, Horswell S, Stewart A, Matthews N, Treisman R (2016). SRF co-factors control the balance between cell proliferation and contractility. Mol. Cell.

[23] Miralles F, Posern G, Zaromytidou AI, Treisman R (2003). Actin dynamics control SRF activity by regulation of its coactivator MAL. Cell.

[24] Rehklau K, Gurniak CB, Conrad M, Friauf E, Ott M, Rust MB (2012). ADF/cofilin proteins translocate to mitochondria during apoptosis but are not generally required for cell death signaling. Cell Death Differ..

[25] Seiler A (2008). Glutathione peroxidase 4 senses and translates oxidative stress into 12/15-lipoxygenase dependent- and AIF-mediated cell death. Cell Metab..

[26] Hinojosa LS, Holst M, Baarlink C, Grosse R (2017). MRTF transcription and Ezrin-dependent plasma membrane blebbing are required for entotic invasion. J. Cell Biol..

[27] Michaelsen-Preusse K, Zessin S, Grigoryan G, Scharkowski F, Feuge J, Remus A, Korte M (2016). Neuronal profilins in health and disease: Relevance for spine plasticity and Fragile X syndrome. Proc. Natl. Acad. Sci. U.S.A..

[28] Baker JM, Boyce FM (2014). High-throughput functional screening using a homemade dual-glow luciferase assay. J. Vis. Exp..

[29] Grohm J, Plesnila N, Culmsee C (2010). Bid mediates fission, membrane permeabilization and peri-nuclear accumulation of mitochondria as a prerequisite for oxidative neuronal cell death. Brain Behav. Immun..

[30] Zhang W, Wu Y, Du L, Tang DD, Gunst SJ (2005). Activation of the Arp2/3 complex by N-WASP is required for actin polymerization and contraction in smooth muscle. Am. J. Physiol..

[31] Korobova F, Ramabhadran V, Higgs HN (2013). An actin-dependent step in mitochondrial fission mediated by the ER-associated formin INF2. Science.

[32] Hoffmann L, Waclawczyk MS, Hanschmann EM, Gellert M, Rust MB, Culmsee C (2020). Cofilin1 oxidation links oxidative distress to mitochondrial demise and neuronal cell death. bioRxiv.

[33] Landshamer S (2008). Bid-induced release of AIF from mitochondria causes immediate neuronal cell death. Cell Death Differ..

[34] Neitemeier S (2017). BID links ferroptosis to mitochondrial cell death pathways. Redox Biol..

[35] Plessner M, Grosse R (2015). Extracellular signaling cues for nuclear actin polymerization. Eur. J. Cell Biol..

[36] Treisman R (2013). Shedding light on nuclear actin dynamics and function. Trends Biochem. Sci..

[37] Morton WM, Ayscough KR, McLaughlin PJ (2000). Latrunculin alters the actin-monomer subunit interface to prevent polymerization. Nat. Cell Biol..

[38] Tsuji T, Miyoshi T, Higashida C, Narumiya S, Watanabe N (2009). An order of magnitude faster AIP1-associated actin disruption than nucleation by the Arp2/3 complex in lamellipodia. PLoS ONE.

[39] McGee KM, Vartiainen MK, Khaw PT, Treisman R, Bailly M (2011). Nuclear transport of the serum response factor coactivator MRTF-A is downregulated at tensional homeostasis. EMBO Rep..

[40] Chaudhry F, Guerin C, von Witsch M, Blanchoin L, Staiger CJ (2007). Identification of Arabidopsis cyclase-associated protein 1 as the first nucleotide exchange factor for plant actin. Mol. Biol. Cell.

[41] Moriyama K, Yahara I (2002). Human CAP1 is a key factor in the recycling of cofilin and actin for rapid actin turnover. J. Cell Sci..

[42] Nomura K, Ono K, Ono S (2012). CAS-1, a *C. elegans* cyclase-associated protein, is required for sarcomeric actin assembly in striated muscle. J. Cell Sci..

[43] Cenik BK, Liu N, Chen B, Bezprozvannaya S, Olson EN, Bassel-Duby R (2016). Myocardin-related transcription factors are required for skeletal muscle development. Development.

[44] Li S (2005). Requirement for serum response factor for skeletal muscle growth and maturation revealed by tissue-specific gene deletion in mice. Proc. Natl. Acad. Sci. U.S.A..

[45] Cotton M, Claing A (2009). G protein-coupled receptors stimulation and the control of cell migration. Cell. Signal..

[46] Moustakas A, Heldin CH (2008). Dynamic control of TGF-beta signaling and its links to the cytoskeleton. FEBS Lett..

[47] Schiller MR (2006). Coupling receptor tyrosine kinases to Rho GTPases–GEFs what's the link. Cell. Signal..

[48] Jaffe AB, Hall A (2005). Rho GTPases: Biochemistry and biology. Annu. Rev. Cell Dev. Biol..

[49] Ohashi K, Nagata K, Maekawa M, Ishizaki T, Narumiya S, Mizuno K (2000). Rho-associated kinase ROCK activates LIM-kinase 1 by phosphorylation at threonine 508 within the activation loop. J. Biol. Chem..

[50] Ono S (2013). The role of cyclase-associated protein in regulating actin filament dynamics—More than a monomer-sequestration factor. J. Cell Sci..

