# Supplementary material for: Cyclase-associated protein 2 (CAP2) controls MRTF-A localization and SRF activity in mouse embryonic fibroblasts
Source: Sci Rep. 2021 Feb 26;11:4789. doi: 10.1038/s41598-021-84213-w (PMC7910472; doi:10.1038/s41598-021-84213-w)
Supplement: Supplementary file 1 — Supplementary Information. [file 41598_2021_84213_MOESM1_ESM.pdf]

Supplementary information

Cyclase-associated protein 2 (CAP2) controls MRTF-A localization and SRF activity in mouse embryonic fibroblasts

Lara-Jane Kepser[1,2,*], Sharof Khudayberdiev[1,*], Laura Soto Hinojosa[2,3], Chiara Macchi[4], Massimiliano Ruscica[4], Elena Marcello[4], Carsten Culmsee[5,6], Robert Grosse[2,3], Marco B. Rust[1,2,6,7]

[1]Institute of Physiological Chemistry, University of Marburg, 35032 Marburg, Germany; [2]DFG Research Training Group, Membrane Plasticity in Tissue Development and Remodeling, GRK 2213, Philipps-University of Marburg, 35032 Marburg, Germany; [3]Institute of Pharmacology, University of Marburg, 35032 Marburg, Germany; [4]Department of Pharmacological and Biomolecular Sciences, University of Milan, 20133 Milan, Italy; [5]Institute of Pharmacology and Clinical Pharmacy, University of Marburg, 35032 Marburg, Germany; [6]Center for Mind, Brain and Behavior (CMBB), University of Marburg and Justus-Liebig-University Giessen, Hans-Meerwein-Strasse 6, 35032 Marburg, Germany; *both authors contributed equally to this work

[7]Correspondence to

Marco B. Rust

Institute of Physiological Chemistry

Philipps-University of Marburg

35032 Marburg, Germany

Phone: +49-6421-2865020

E-Mail: marco.rust@staff.uni-marburg.de

Legends to supplementary figures and movies

Figure S1. Impaired actin turnover in CAP2-KO MEFs. Representative micrographs from GFP-actin transfected CTR MEF (upper row, also see Movie S1), KO MEF (middle row, also see Movie S2) and CTR MEF treated with 200 nM JASP (lower row, also see Movie S3) during selected time-points of FRAP assay. Bleached region (dotted circle) of cell cytoplasm has been indicated in the first column (pre-bleaching). Scale bars represents 10 µm.

Figure S2. Full length immunoblots. Original immunoblots and respective biological replicates (indicated as N1-N5) from Fig. 1A, F and G are given. Lysates from CTR cells are depicted as 202C and 202D; and lysates of KO cells as 201C and 201F. Cropped micrographs used in the main figure are indicated with black dotted rectangles.

Figure S3. CAP2 inactivation does not affect energy metabolism and mitochondrial resilience in MEFs. (A) Real-time cell impedance measurements performed in CTR and KO MEFs. Cells were treated or not with erastin (0.7 µM). **(B)** Metabolic activity was determined by MTT assay. CTR and KO MEFs were challenged or not with erastin (0.7 µM) for eight hours and were presented as percentage of CTR. **(C)** Representative micrographs of CTR and KO MEFs stained with MitoTracker Deep Red to visualize mitochondrial morphology. Scale bars: 25 µm. **(D)** Analysis of mitochondrial fragmentation performed in five hundred CTR and KO MEF cells quantified as percentage of counted cells. **(E)** MitoSOX staining and subsequent FACS analysis were performed in CTR and KO MEFs treated or not with erastin (0.7 µM) for eight hours. **(F)** BODIPY 581/591 staining and subsequent FACS analysis were performed in CTR and KO MEFs treated or not with erastin (0.7 µM) for eight hours. **(G)** TMRE staining and subsequent FACS analysis were performed in CTR and KO MEFs treated or not with erastin (0.7 µM) for sixteen hours. For panels E, F and G, data are presented as percentage of gated cells and are representative of three replicates per condition.

Figure S4. CAP2 localizes in the cytoplasm of MEF cells. Representative micrographs showing localization of GFP-tagged CAP2 (white or green) in CTR MEFs in basal (1st row), starved (2nd row) and serum stimulated conditions (3rd and 4th rows). Merge micrograph (right panel) includes counterstaining with DNA dye Hoechst (blue).

Movie S1. Representative movie of GFP-actin-transfected CTR MEF during FRAP experiment.

Movie S2. Representative movie of GFP-actin-transfected KO MEF during FRAP experiment.

Movie S3. Representative movie of GFP-actin-transfected CTR MEF during FRAP experiment. MEFs were treated with 200 nM JASP for 1 h.

Movie S4. Representative movie of serum-stimulated CTR MEFs that stably express GFP-tagged MRTF-A. Label '+ serum' indicates time point when 20% FCS was added to the medium.

Movie S5. Representative movie of serum-stimulated KO MEFs that stably express GFP-tagged MRTF-A. Label '+ serum' indicates time point when 20% FCS was added to the medium.

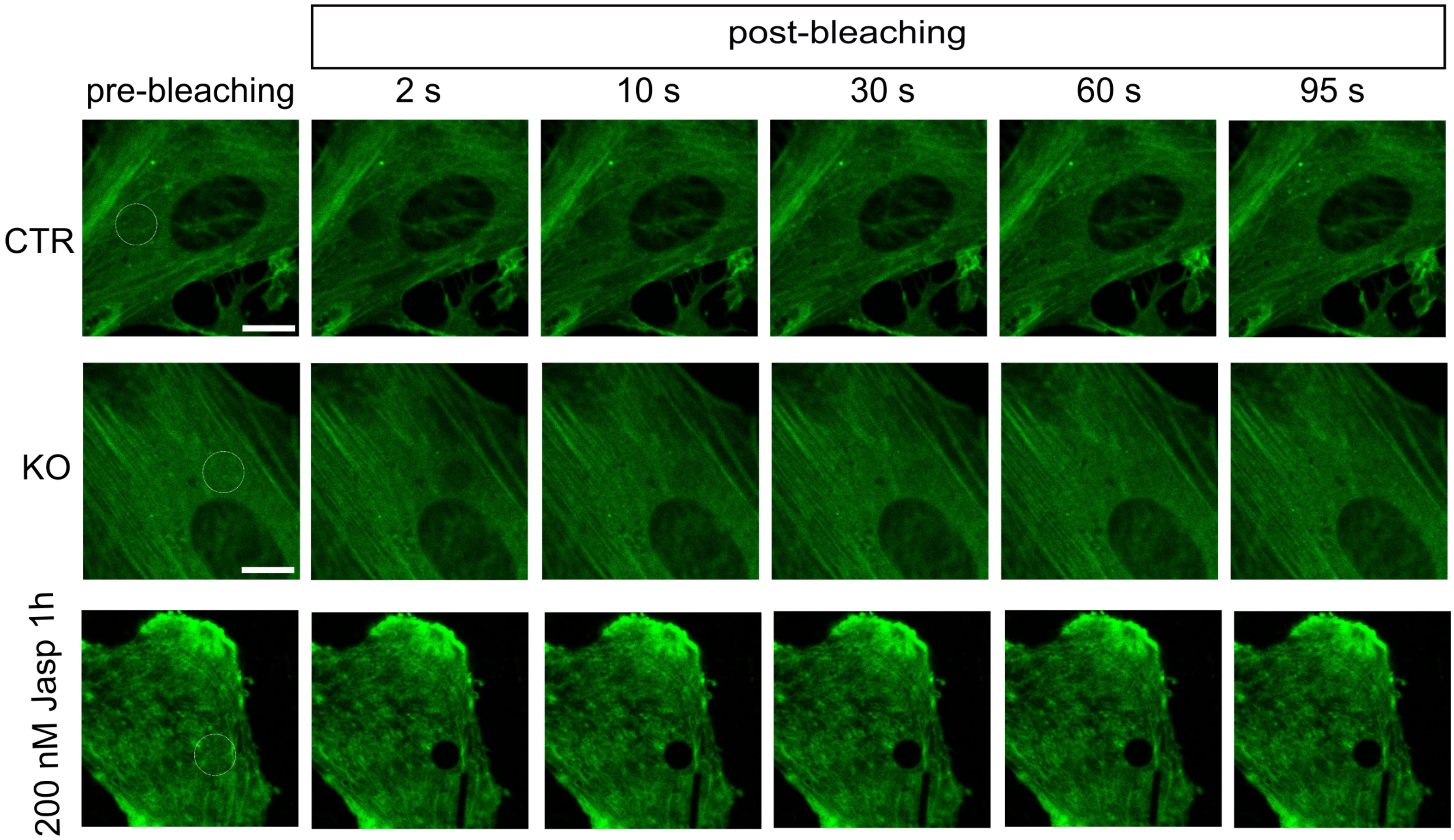
post-bleaching
pre-bleaching
2 s
10 s
30 s
60 s
95 s
CTR
KO
200 nM Jasp 1h

Figure S1

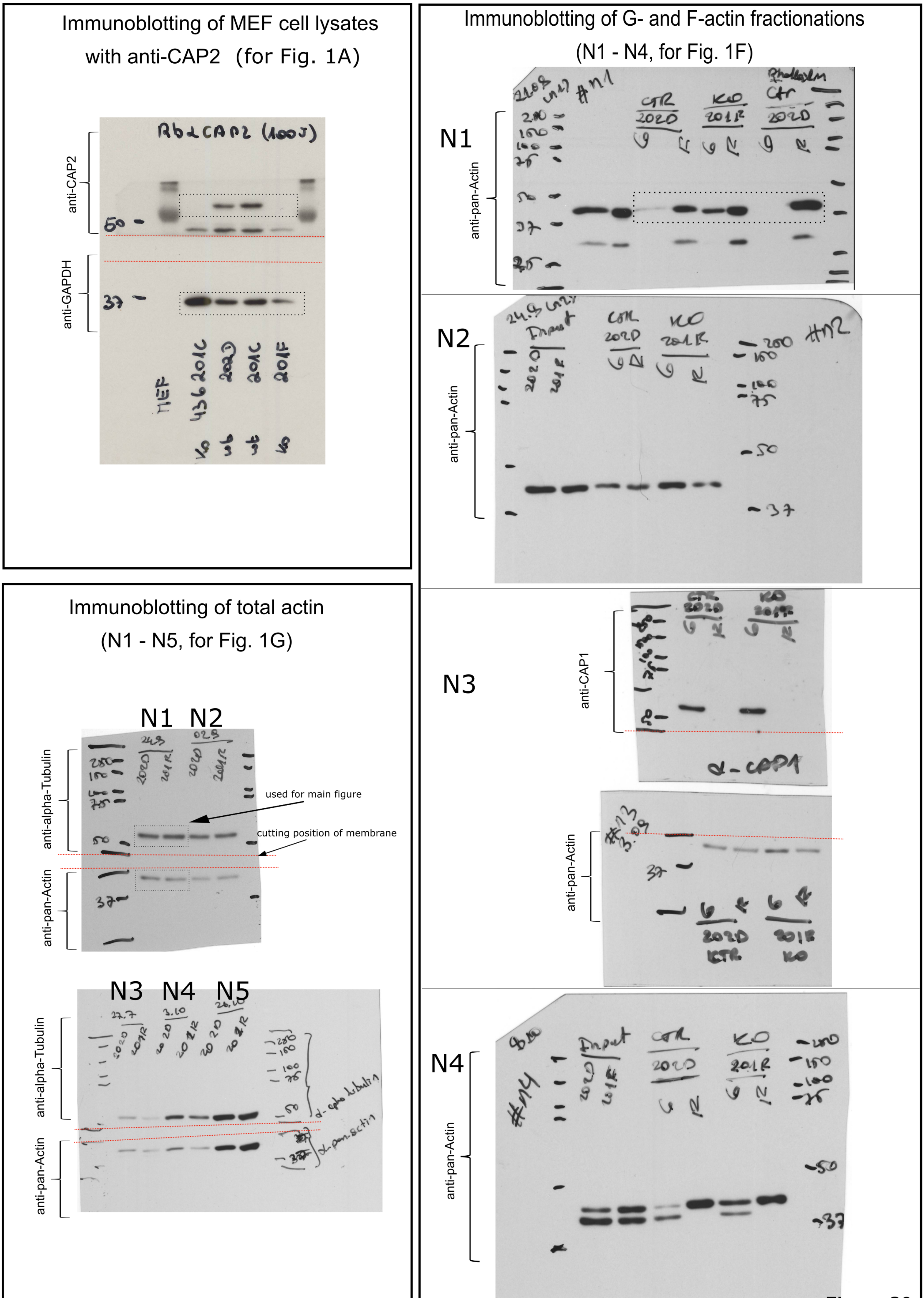
Immunoblotting of MEF cell lysates with anti-CAP2 (for Fig. 1A)
anti-CAP2
anti-GAPDH
Immunoblotting of total actin (N1 - N5, for Fig. 1G)
N1
N2
anti-alpha-Tubulin
anti-pan-Actin
used for main figure
cutting position of membrane
N3
N4
N5
anti-alpha-Tubulin
anti-pan-Actin
Immunoblotting of G- and F-actin fractionations (N1 - N4, for Fig. 1F)
N1
anti-pan-Actin
N2
anti-pan-Actin
N3
anti-CAP1
anti-pan-Actin
N4
anti-pan-Actin

Figure S2

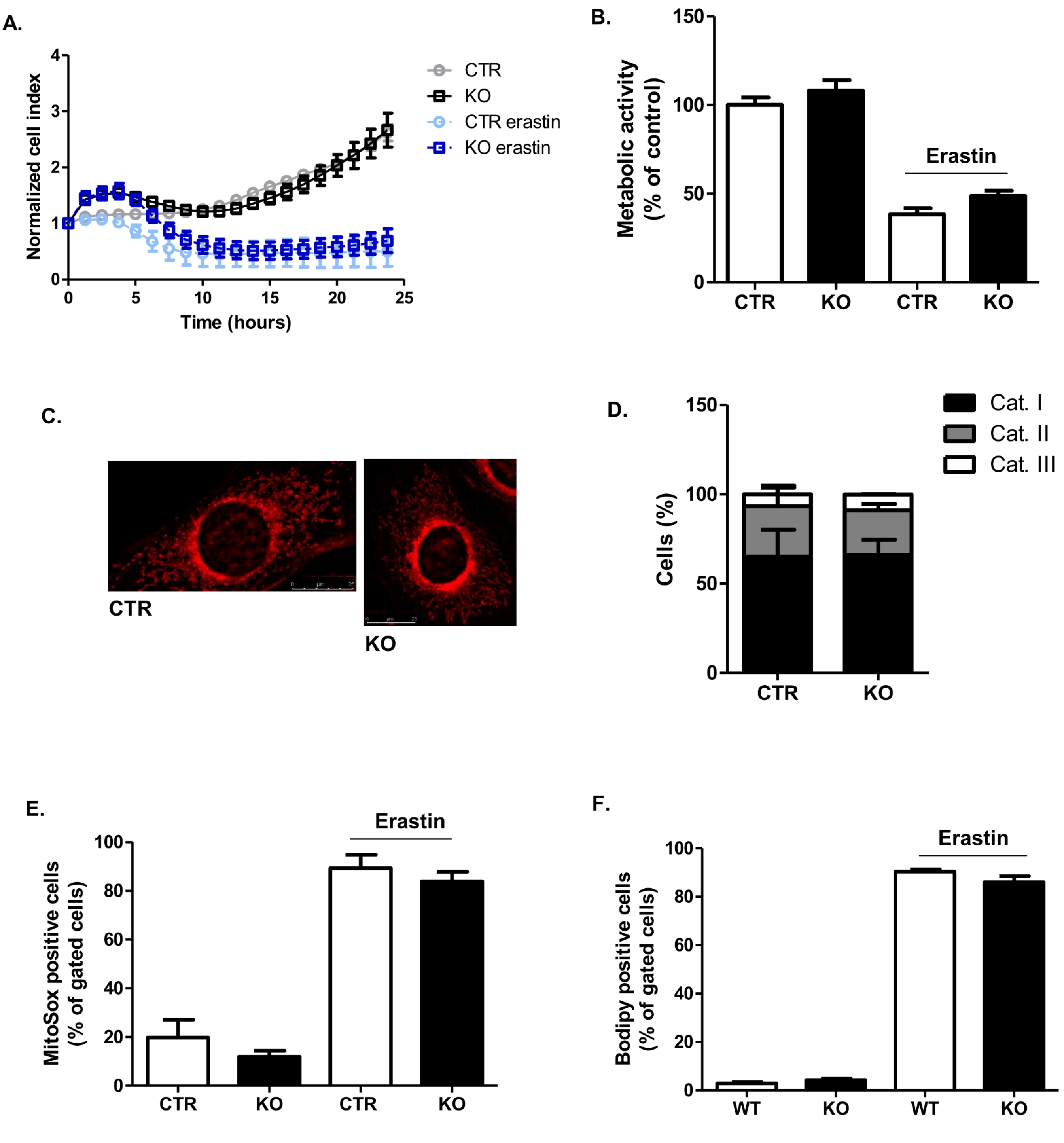
A.
Normalized cell index
Time (hours)
CTR
KO
CTR erastin
KO erastin
B.
Metabolic activity (% of control)
Erastin
CTR
KO
CTR
KO
C.
CTR
KO
D.
Cells (%)
Cat. I
Cat. II
Cat. III
CTR
KO
E.
MitoSox positive cells (% of gated cells)
Erastin
CTR
KO
CTR
KO
F.
Bodipy positive cells (% of gated cells)
Erastin
WT
KO
WT
KO

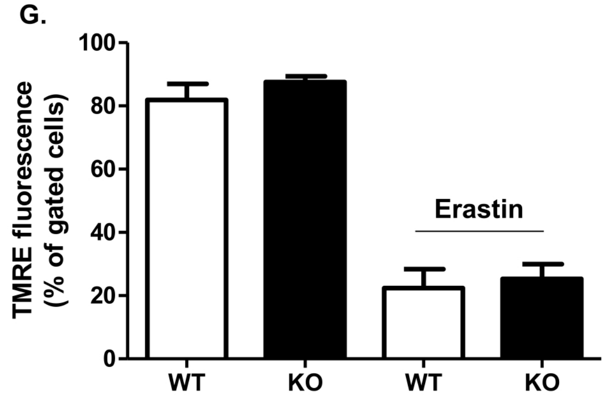
G.
TMRE fluorescence (% of gated cells)
Erastin
WT
KO
WT
KO

Figure S3

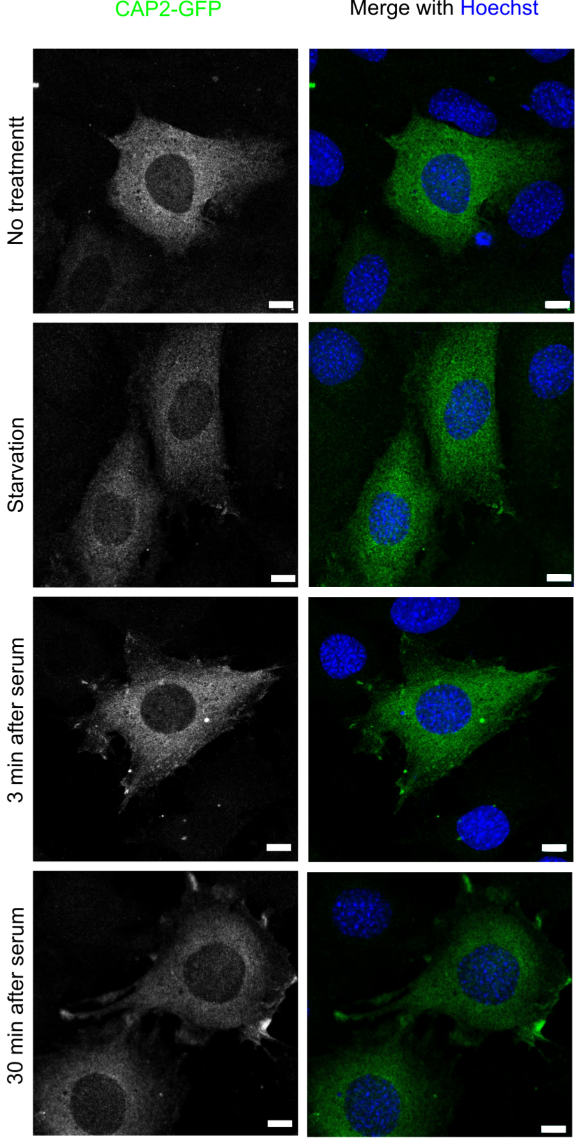
CAP2-GFP
Merge with Hoechst
No treatmentt
Starvation
3 min after serum
30 min after serum

Figure S4
